# *In Silico* Structural Homology Modelling and Docking for Assessment of Pandemic Potential of a Novel H7N9 Influenza Virus and Its Ability to Be Neutralized by Existing Anti-Hemagglutinin Antibodies

**DOI:** 10.1371/journal.pone.0102618

**Published:** 2014-07-21

**Authors:** Harinda Rajapaksha, Nikolai Petrovsky

**Affiliations:** 1 Vaxine Pty Ltd, Bedford Park, Adelaide, South Australia, Australia; 2 Department of Diabetes and Endocrinology, Flinders Medical Centre/Flinders University, Adelaide, South Australia, Australia; 3 Vaxine Pty Ltd, Flinders Medical Centre/Flinders University, Adelaide, South Australia, Australia; The University of Hong Kong, China

## Abstract

The unpredictable nature of pandemic influenza and difficulties in early prediction of pandemic potential of new isolates present a major challenge for health planners. Vaccine manufacturers, in particular, are reluctant to commit resources to development of a new vaccine until after a pandemic is declared. We hypothesized that a structural bioinformatics approach utilising homology-based molecular modelling and docking approaches would assist prediction of pandemic potential of new influenza strains alongside more traditional laboratory and sequence-based methods. The newly emerged Chinese A/Hangzhou/1/2013 (H7N9) influenza virus provided a real-life opportunity to test this hypothesis. We used sequence data and a homology-based approach to construct a 3D-structural model of H7-Hangzhou hemagglutinin (HA) protein. This model was then used to perform docking to human and avian sialic acid receptors to assess respective binding affinities. The model was also used to perform docking simulations with known neutralizing antibodies to assess their ability to neutralize the newly emerged virus. The model predicted H7N9 could bind to human sialic acid receptors thereby indicating pandemic potential. The model also confirmed that existing antibodies against the HA head region are unable to neutralise H7N9 whereas antibodies, e.g. Cr9114, targeting the HA stalk region should bind with high affinity to H7N9. This indicates that existing stalk antibodies initially raised against H5N1 or other influenza A viruses could be therapeutically beneficial in prevention and/or treatment of H7N9 infections. The subsequent publication of the H7N9 HA crystal structure confirmed the accuracy of our *in-silico* structural model. Antibody docking studies performed using the H7N9 HA crystal structure supported the model's prediction that existing stalk antibodies could cross-neutralise the H7N9 virus. This study demonstrates the value of using *in-silico* structural modelling approaches to complement physical studies in characterization of new influenza viruses.

## Introduction

One of the leading challenges when a new influenza strain such as H7N9 is found to be infecting humans is to rapidly appraise its pandemic potential, so as to gauge the importance of allocation of resources to study of the new virus and development of tools and reagents including vaccines [1], [2]. Bottlenecks in pandemic assessment arise from delays in transporting the new virus to laboratories with the requisite skills, the time to grow and characterise the virus, infect animals and develop an understanding of its behaviour [1]. This process may take 6–12 months, with development of a vaccine against the new strain taking potentially even longer [3]. Advances in bioinformatics including structural modelling and docking tools provide a major opportunity to help assess potential pandemic influenza viruses alongside their physical characterisation [4]. Ultimately, this could assist decisions to commence pandemic preparations including vaccine production and thereby ensure faster pandemic vaccine supply. Key questions that could potentially be addressed by structural modelling methods to help assess the pandemic potential of any new influenza virus include capability of human to human transmission, ability to acquire mutations that could increase virulence, ability to be neutralised by existing antibodies and/or antiviral drugs and suitability for egg or cell culture adaptation and large-scale vaccine production [4].

The human outbreak in China in February 2013 of respiratory infections due to a novel avian-origin influenza A/Hangzhou/1/2013 (H7N9) virus [5], [6] allowed a unique opportunity for a live-fire exercise to test the latest structural modelling approaches to study the newly isolated H7N9 virus and predict its pandemic potential in parallel with its laboratory characterisation. In the first few weeks after publication of the initial H7N9 sequence, analyses were done by sequence alignment analysis, with specific amino acid mutations identified within H7N9 that could potentially predict human adaptation and pandemic potential [7]–[10]. A study based on H7N9 sequence analysis reported a low number of predicted T-cell epitopes potentially signifying low vaccine immunogenicity [11]. Although useful, sequence analysis is a qualitative rather than quantitative tool and does not allow precise estimates of human receptor binding affinity, a key element in assessment of pandemic potential. Additional analyses utilising structural models could allow much more accurate prediction of receptor binding affinity and at the same time could provide an unique opportunity to test the ability of existing human antibodies to bind and neutralise the new viral isolate [12]. We therefore asked in this study whether a structural modelling approach focussed on building a structural homology model of H7 hemagglutinin (HA) followed by docking studies with host receptors and potential neutralizing antibodies could assist assessment of the pandemic potential of novel avian influenza strains such as H7N9.

## Methods

### Homology modelling

The study as depicted in the flow diagram in Figure S1 in file S1 was performed in late May 2013. As a first step, the amino acid sequence of the HA protein of A/Hangzhou/1/2013H7N9, Accession AGI60301 (Hangzhou-H7) [13] was submitted to Swiss-model workplace [14] and subjected to Gapped Blast using BLOSUM 62 matrix with an E-value cut-off of 0.000001 to identify the closest homologous structure as at that date. The closest template 4DJ8.PDB [15] representing the crystal structure of A/Netherlands/219/2003 (H7N7) was aligned with the Hangzhou-H7 sequence using Modeller v9.11 [16] alignment script. The 3D homology model of Hangzhou-H7 was created with the Modeller v9.11 [16] automodel class using the alignment as a guide. Then Discreet Optimised Protein Energy Score (DPOES) based model selection and refinements were conducted using Modeller v9.11 [16] evaluation and loop refining scripts. Finally, the model was relaxed using the molecular dynamics program NAMD v2.9 [17] for 1000 steps at 1-time steps at 310 K temperature. The final Hangzhou-H7 structure model validation was conducted with ProSA [18] and QMEAN servers [19]. The packing quality of each model was evaluated using the ANOLEA server [20].

### Docking glycoprotein-linked sialic acid receptors to Hangzhou-H7 structure model

Glycan receptors can undergo conformational changes during the binding process to different HA types [21]. Hence use of a known co-crystallised glycan receptor-HA structure for the glycan structure to be used in the proposed docking study could introduce a favoured receptor conformation, thereby biasing the results. To avoid such a potential bias we constructed and energy minimised each glycan receptor structure prior to testing its docking to HA. The 3 D structures of α2,3-linked sialic acid and α2,6-linked sialic acid were constructed using the Visual Molecular Dynamics v1.9.1 (VMD) [22] plugin molefacture and the structures were minimised using AMBER [23] force field parameters. Then each glycoprotein-linked sialic acid molecule was docked to the Hangzhou-H7 structure model using Autodock Vina v1.12 [24] and binding energies were calculated.

### Docking neutralizing antibodies to Hangzhou-H7 structure model

3 D coordinate files of known crystal structures neutralizing antibodies in complex with various HA molecules were obtained from Protein Data Bank (PDB) and their antigen-binding complementarity determining region (CDR)-3 regions were separated from the complex using the molecular visualizing and editing program UCSF Chimera v1.7 [25]. Based on structural alignments between Hangzhou-H7 and HA neutralizing antibody complexes those respective regions on Hangzhou-H7 globular head or stalk were docked on to the (CDR)-3 region of the corresponding neutralizing antibody, using Autodock v4.2.5.1 [26]. For the docking calculation, the (CDR)-3 region was selected as the receptor and antigen binding loops were set as flexible while the ligand was considered rigid. The docking analysis was conducted with multiple replications and each time the ligand was positioned on a randomly determined place on a virtual 3 D grid using a random number. Then using Monte Carlo simulated annealing different binding modes were simulated and binding energies for each mode were calculated. Finally each binding mode was scored using the scoring function available in Autodock and the best binding mode with the corresponding binding energy was identified. Docking was conducted using the high performance computer available at Flinders University, South Australia.

## Results

### Structural Model of Hangzhou-H7

Influenza A viruses are subdivided into group 1 (H1, H2, H5, H6, H8, H9, H11, H12, H13, H16 and H17) and group 2 (H3, H4, H7, H10, H14 and H15) strains based on surface hemagglutinin (HA) subtypes. At the time this project was initially undertaken no H7N9 HA crystal structure was available. In order to characterise the behaviour of H7N9 HA a homology model was generated. The generated models were validated for their packing quality by evaluating the “Non-Local Environment” (NLE) of each heavy atom in the molecule (ANOLEA) [20]. The global quality each chain in the model was assessed by calculating the QMEAN scores using the QMEAN server [19] and comparing the model with experimentally determined X-ray and NMR structures using the ProSA server [18]. ANOLEA [20] packing quality analysis of HA1 and HA2 chains in the model demonstrated 95% negative energy values for HA1 and 93% for HA2 (Figure S2 in file S1), which indicated a high packing quality within the model. The QMEAN [19] global quality score for HA1 (0.79 out of 1 and the Z-score of 0.13) indicated that the model for HA1 was of high quality but QMEAN [19] of 0.49 and Z-score of -3.62 for the HA2 model suggested a lower but acceptable quality model (due to the presence of hydrophobic α helix regions which cause lower QMEAN score and the Z-score (Figure S3 in file S1) [19]. The models of both chains were compared against experimentally determined X-ray and NMR structures using the ProSA server [18] and their global and local quality was scored. Based on the ProSA [18] analysis, both HA1 and HA2 chains were within the acceptable quality for the overall model quality with Z-scores −8.06 and −2.42, respectively (Figure S4A in file S1). Plotting the energy values of each amino acid as a function of the sequence presented negative knowledge based energies for all the amino acids of the HA1 chain (Figure S4B in file S1), reiterating its high quality. However, for the HA2 chain, those amino acids located within the loop regions presented positive energy values, most likely due to the presence of amino acids with the capacity of forming a helix in a loop. Thus both HA1 and HA2 models were within the acceptable range and closely resembled homologous crystal structures.

### Structure of Hangzhou-H7 and affinity for human and avian receptors

**Figure 1:**
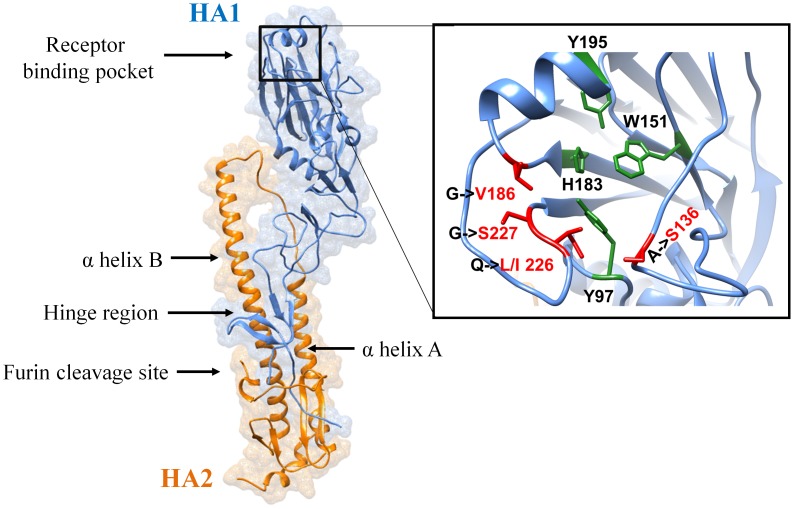
Structural model of H7N9. The homology model of Hangzhou-H7 built using the 4DJ8.PDB H7N7 crystal structure as a template. The zoomed region shows the receptor-binding pocket. Residues that form the receptor-binding pocket are highlighted in green and those mutations that determine receptor specificity are highlighted in red.

The overall predicted structure of Hangzhou-H7 closely resembled other reported HA structures (Figure 1). HA is synthesized as a single chain precursor and then cleaved into HA1 and HA2 by furin, a host enzyme [27]. The HA1 contains the globular head like structure located at the membrane distal end of the molecule. The HA2 is located at the membrane proximal end and has a distinctive central helical stalk with two distinct helixes (αA and αB) [27] that facilitate membrane fusion during infection [27]–[29]. The HA receptor-binding site, which interacts with glycoprotein-linked sialic acid residues and anchors the virus particle on respiratory tract cell membranes, is found within the globular head of HA1 [27]. The receptor-binding pocket on Hangzhou-H7 is made of four residues (Y98, W152, H184 and Y196) [30], highlighted in green in Fig. 1. Sequence analysis previously suggested that the T138A, G187V, Q226I and G227S mutations located within the receptor-binding pocket of Hangzhou-H7 may enhance its affinity for the human sialic acid receptor, thereby reflecting pre-existing mammalian adaptation [7].

**Figure 2:**
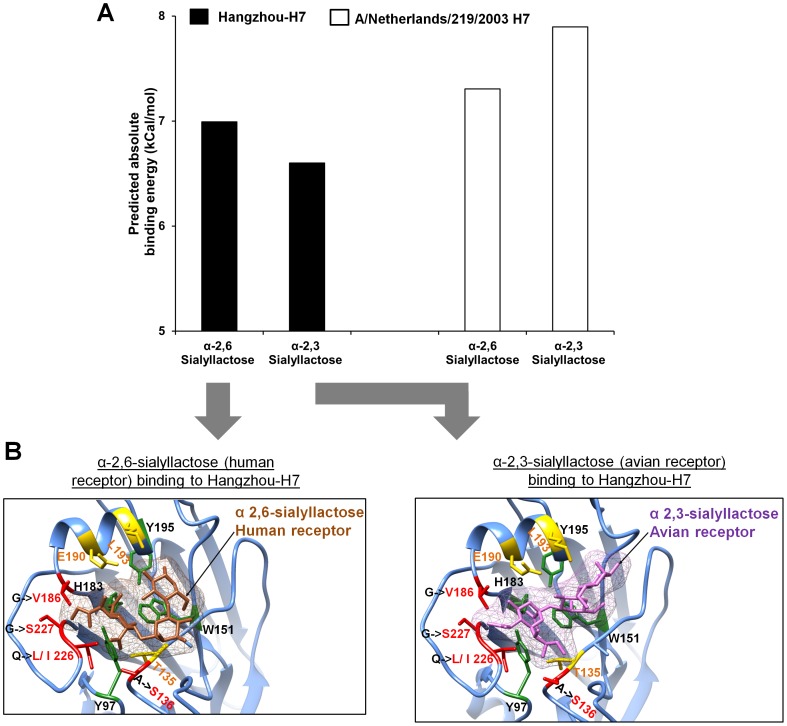
Binding affinity of Hangzhou-H7 and H7N7 for human and avian receptors. Hangzhou-H7 and the closely related H7N7 virus (A/Netherlands/219/2003) were docked to human and avian sialic acid receptors and affinities calculated. (**A**) The predicted Hangzhou-H7 model receptor affinities are shown by the black bars and the A/Netherlands/219/2003 affinities by the white bars. (**B**) The highest affinity docking conformation of Hangzhou-H7 on the human (brown), and avian (purple), receptors. HA mutations that influence the receptor binding specificity are highlighted in red and other residues that interact with the receptor in yellow. Residues that form the receptor-binding pocket are highlighted in green.

To better assess the precise implication of these mutations on Hangzhou-H7's ability to bind avian and human sialic acid receptors, the structural model was used to perform docking studies and thereby measure respective receptor-binding affinities. The affinities of Hangzhou-H7 for human and avian receptors were measured after docking the corresponding receptor to Hangzhou-H7 receptor binding pocket using Autodock vina v1.12 [24]. For comparison, a high pathogenicity avian H7N7 strain (structure 4DJ8.PDB) [15], which has a glutamine at position 226 (Q226) instead of the Isoleucine (I226) for H7N9 was also docked to the same receptors. The docking analysis measured affinities for 9 different binding modes of HA for each receptor (Fig. 2). H7N7 that possesses Q226 had marginally higher affinity for the avian (−7.9 kcal/mol) than the human receptor (−7.3 kcal/mol) whereas Hangzhou-H7 that possesses I226 had a marginally higher predicted affinity for the human (−6.9 kcal/mol) than the avian receptor (−6.6 kcal/mol). This observation is supported by mutation studies that demonstrated an increased affinity of a H7 HA for human receptor after Q226L, which is similar to Q226I mutation [31]. This indicates that Hangzhou-H7 when compared to H7N7 HA is better adapted for binding to the human receptor, increasing the potential for it to infect humans and become a pandemic strain.

### Antibody neutralization of Hangzhou-H7

**Figure 3:**
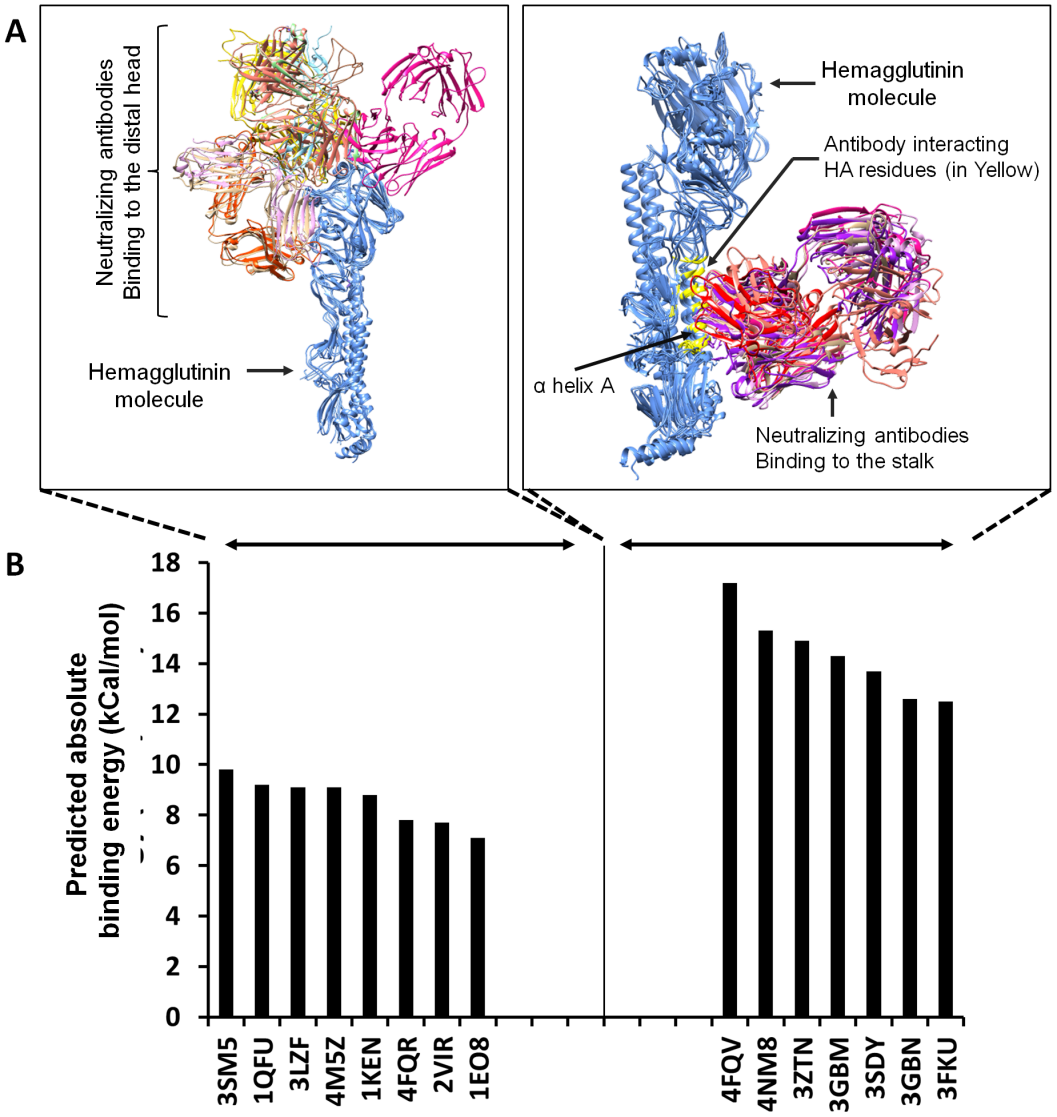
Hangzhou-H7 neutralizing antibody binding. (**A**) The structures of neutralizing antibodies are shown either binding to the Hangzhou-H7 (shown in blue) globular head region (left figure) or stalk region (right figure). Different colours represent different antibodies. HA residues interacting with stalk-binding neutralizing antibodies are shown in the right hand figure highlighted in yellow. (B) HA-neutralizing antibodies binding the head or stalk regions were docked to the predicted Hangzhou-H7 structure and binding energies were calculated and presented as an absolute value. PDB IDs of antibodies against H1 are 3LZF, 3SM5, 4M5Z, 3GBN, 3ZTN, PDB IDs for H3 are 1EO8, 1KEN, 1QFU, 2VIR, 4FQR, 3SDY, 4NM8, H5 PDB IDs are 3FKU and 3GBM and H7 ID is 4FQV.

Sequence analysis has shown that the HA head region is subject to a high level of mutation of residues in the receptor-binding pocket. These mutations act to maintain virus HA receptor binding specificity for host sialic acid receptors but to reduce binding affinity of existing neutralising antibodies, thereby allowing viral escape [31]. Over the years, humans are exposed to HA variants from natural virus infections or through immunization and build up a complex antibody repertoire against different virus strains as a result. Therefore, we used currently available crystal structures of H1, H3, H5 and H7 HA-antibody complexes to predict which existing antibodies might be capable of neutralizing Haghzhou-H7 [13]. Querying for HA-human antibody complexes at PDB produced 15 structures of unique HA-antibody complexes with antibodies bound to either the HA head (8 complexes) or stalk (7 complexes) region. Within the set of HA head-binding antibodies for which structures existed, five antibodies were against H3 (corresponding PDB identifiers: 1EO8 [32], 1KEN [33], 1QFU [34], 2VIR [35], and 4FQR [36]) and three were against H1 (corresponding PDB identifiers: 3LZF [37], 3SM5 [38] and 4M5Z [39]). Within the HA stalk-binding antibodies, two antibodies against H5 (corresponding PDB identifiers: 3FKU [40] and 3GBM [41]), two against H3 (corresponding PDB identifiers: 3SDY [42] and 4NM8 [43]), two against H1 (3GBN [41] and 3ZTN [44]) and one against H7 (corresponding PDB identifier: 4FQV [45]). Using UCSF Chimera [25] to measure the van der Waals (VDW) radii overlap between the atoms of the antibody and the HA, the residues on HA that function as contact sites for each antibody were identified. Those neutralizing antibodies that bind to the head region and thereby prevent HA interaction with its sialic acid receptor [27] recognize a number of different sites while neutralizing antibodies that bind the HA stalk and inhibit pH dependent conformational HA changes and membrane fusion [28], [46] specifically recognize αA helix and HA1 neighbouring residues (Figure 3A). To analyse the potential neutralizing capability of previously described neutralizing antibodies they were docked to the stalk or the head of Hangzhou-H7 using Autodock v4.2.5.1. The antibody Cr9114 that was previously crystallised with H7N7 strain A/Netherlands/219/2003 (4FQV) was predicted to bind the Hangzhou-H7 stalk region with the highest affinity, although all the stalk antibodies irrespective of the initial strain against which they were raised or crystallised were predicted to bind Hangzhou-H7 with higher affinity than the HA head-binding antibodies analysed and thereby could possess the ability to neutralise H7N9 (Figure 3B). By contrast, all the neutralising antibodies known to bind the HA head region were predicted to bind Hangzhou-H7 with relatively low affinity, suggesting a low probability of their neutralising H7N9 (Figure 3B).

**Figure 4:**
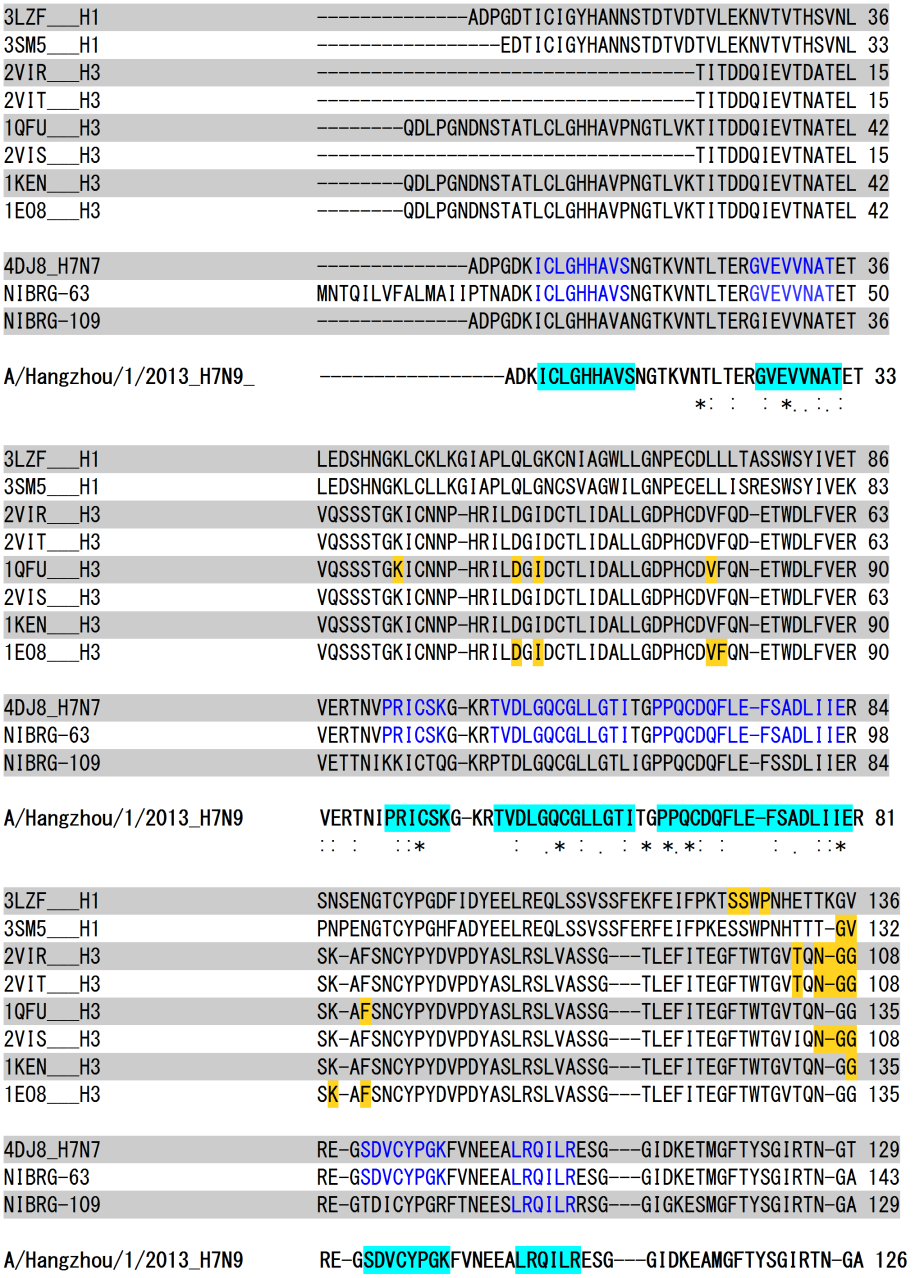
Multiple sequence alignment of HA1 of crystalized antibody-HA complexes of H1, H3, A/Netherlands/219/2003(H7N7) (4DJ8) and WHO H7N9 vaccine candidates (NIBRG-63 and NIBRG-109). Residues on HA1 which are within 4 Å distance from the docking site of the neutralizing antibody are highlighted in yellow on the respective HA sequence available on the crystalized antibody-HA complex. Linear antigenic epitopes, as predicted by using *Kolaskar* and *Tongaonkar antigenicity* scale on Hangzhou/H7 are highlighted in cyan and homologous regions to those epitopes on A/Netherlands/219/2003(H7N7) and WHO vaccine candidates (NIBRG-63 and NIBRG-109) are highlighted in blue.

To address the question of whether neutralising head antibodies generated by seasonal influenza vaccines are likely to be able to neutralise the H7N9 virus, we performed a multiple sequence alignment of HA1 of crystalized antibody-HA complexes of H1 (2 sequences), H3 (6 sequences) the HA1 of highly-pathogenic A/Netherlands/219/2003(H7N7) (4DJ8) and two of the World Health Organisation (WHO) recommended H7 vaccine candidates (NIBRG-63 and NIBRG-109). As shown in Figure 4, multiple amino acids on HA which are recognized by H1 and H3 neutralizing antibodies have been mutated in H7N9 providing it with a means of escape from neutralization by existing antibodies to seasonal vaccine strains. By contrast, A/Netherlands/219/2003(H7N7) and the WHO vaccine candidates NIBRG-63 and NIBRG-109 demonstrate a high sequence homology to H7N9 HA (92.7%, 96.5% and 76.6% respectively) and have similar predicted antigenic epitopes. A/Netherlands/219/2003 and NIBRG-63 share seven antigenic HA epitopes with H7N9 whereas NIBRG-109 shares only one epitope with 100% identity, consistent with, NIBRG-109 being the most distantly related candidate to H7N9 based on sequence identity.

Following publication of the H7 HA crystal structure, further docking analyses were conducted using the 4BSB.PDB crystal structure using neutralising antibodies previously co-crystallised with H3, H5 and H7 HA to compare to the antibody docking results obtained using the *in-silico* HA homology model. The anti-H3 antibody binds the globular head region and is described in 1EO8.PDB whereas the anti-H5 and anti-H7 antibodies bind the stalk region and are described in 3FKU.PDB and 4FQV.PDB, respectively. The calculated affinity of the anti-H3 head antibody for H7 was ∼0.5 (+/−0.4) kcal/mol lower compared to the already low affinity predicted by the homology model. In contrast, the calculated affinity of the anti-H5 and anti-H7 stalk antibodies for H7 were 0.5 (+/−0.2) kcal/mol higher than the already high binding affinities predicted by the model. These subtle differences in predicted affinity could be a result of the subtle structural differences in side chains positioning between the *in-silico* structural model and the crystal structure. Notably, however the *in-silico* structural model successfully predicted the ability of the existing HA stalk antibodies to neutralise the new H7N9 virus, versus the failure of existing HA head antibodies, with the models predictions being supported by the docking studies using the crystal structure.

## Discussion

This study demonstrates the feasibility and speed of using high performance computing approaches to protein modelling and docking to rapidly generate accurate structural models and predict cellular interactions of key proteins encoded by new influenza strains. This could enable preliminary assessment of pandemic potential of newly emerged influenza strains by providing an estimation of their affinity for human sialic acid receptors and their ability to be neutralized by existing anti-HA antibodies. Use of our structural model of Hangzhou-H7 for docking analysis predicted that it had a similar affinity for the human α2,6 sialyl-lactose receptor (−6.9 kcal/mol) than the avian α2,3 sialyl-lactose receptor (−6.6 kcal/mol), which could suggest increased pandemic potential [21], [47]. When assessed using the same model, the HA of the high pathogenicity avian H7N7 strain showed a higher affinity for the avian than the human receptor, as might be expected of a non-mammalian adapted strain. These findings show structural modelling complements the information obtained by sequence alignment analysis based on comparison with past influenza isolates which identified a number of mutations in the Hangzhou-H7 sequence (A136S, G186V, G227S and Q226L/I) that predicted potential increased binding to the human receptor [48]. Our structure-based analysis demonstrated the significance of these mutations and showed how these mutations change the affinity for the human receptor while retaining affinity for the avian receptor [48]. Such human adaptation has previously been shown to be a critical step in acquisition of pandemic potential [49]. For example, E190D and G225D substitutions in the receptor binding domains of H1N1 and Q226L and G228S substitutions in H2N2/H3N2 were shown to allow avian restricted influenza viruses to bind human receptors and acquire human transmissibility [50]. The H1N1/2009pdm strain that caused the 2009 pandemic was similarly demonstrated using glycan arrays to have acquired high affinity binding to the human receptor while retaining binding to the avian receptor [51], with this array based-study confirming results from an earlier homology-based structural model of H1N1/2009pdm receptor binding affinity which made the same finding [4]. Similarly Q226L and G228S substitutions in the glycan receptor-binding site of H7 HA have been shown to substantially increase its binding affinity to human receptor [31]. This demonstrates the importance of developing both laboratory and computational tools in parallel to assess pandemic potential based on the prediction of the affinity of new influenza isolates for the human receptor.

Another key factor in considering pandemic potential of a new influenza strain is being able to assess the likelihood that antibodies generated by past human influenza infections will be able to neutralize the new virus. Using the Hangzhou-H7 structural modelling approach we were able to assess binding of previously characterised heterologous HA antibodies to the new H7N9 virus. To date, two types of HA neutralizing antibodies have been identified; HA head region antibodies that prevent virus receptor binding and stalk antibodies that prevent the HA conformational changes required for viral fusion [28]. As the HA head region is located most distal to the membrane it is relatively more accessible and hence is the target of most anti-HA antibodies. Unfortunately, given the critical role of the HA head region to receptor binding the influenza virus has evolved strategies to constantly mutate key residues within this region to generate daughter strains that retain receptor binding ability while escaping pre-existing neutralising head antibodies [5]. By contrast, the HA stalk region is located proximal to the membrane and is more inaccessible but those antibodies that bind this region and thereby inhibit virus membrane fusion have been shown to be much more broadly cross-neutralising [52]–[56]. Consistent with these previous findings, our model showed no shared conserved antibody binding motif in the globular head of Hangzhou-H7 and current seasonal influenza strains, thereby predicting that most of the human population will not have significant HA inhibition titers against H7N9 virus. This was confirmed by the fact that known antibodies able to neutralise seasonal or H5 strains were predicted by our docking studies to have a low binding probability for Hangzhou-H7. By contrast, calculation of binding energies for known HA stalk antibodies demonstrated high binding probability in all cases. In particular, the monoclonal antibody Cr9114 [45] that was previously crystallised with H7N7 strain A/Netherlands/219/2003 [15], showed the highest affinity for Hangzhou-H7 of all anti-influenza antibodies we tested. This is an important finding as it suggests that Cr9114 and the other well characterised HA stalk antibodies could potentially be developed as therapeutic antibodies against H7N9, e.g. for use in blocking ongoing viral replication in H7N9 infected patients. The other implication of this finding is that it suggests that vaccines in development based on HA antigens that induce HA stalk antibodies, e.g. ‘headless HA antigens’ should be effective not just against seasonal and H5N1 influenza strains as previously reported [56], [57] but also against the new H7N9 strain.

Subsequent to completion of this study but prior to its publication, the crystal structure of H7N9 HA was published and its ability to bind cells bearing human or avian receptors assessed in laboratory studies [58], [59]. This allowed us to check the accuracy of our structure-based predictions against the experimental data (Figure S5 in file S1). The Hangzhou H7 model aligns with the crystal structure of A/Anhui/1/2013 (H7N9) (4BSB.PDB) [58] with a RMSD of 0.721 Å across 305 atoms, consistent with the predicted structure being an almost identical match to the subsequent H7N9 HA crystal structure (Figure S6 in file S1). Similarly, our prediction of H7N9 HA binding both mammalian and avian receptors was in agreement with measurements of H7N9 virus binding, which demonstrated significant binding to human sialic acid receptors [58]. Furthermore it was shown that H7N9 virus bound to both avian-type and human-type receptors, invaded epithelial cells in the human lower respiratory tract and type II pneumocytes in alveoli, and replicated efficiently in lung and trachea explant cultures and mammalian cell lines [59]. This ability of H7N9 to bind both human and avian receptors is also supported by recent data showing that H7N9 replicated efficiently in the upper and lower respiratory tracts of nonhuman primates and nasal turbinates of ferrets, was able to transmit by respiratory droplet in some ferret pairs and bound to human virus-type receptors on a glycan array [60]. Similarly, H7N9 was shown to infect the upper and lower respiratory tract of ferrets and transmit via direct contact but less efficiently by airborne exposure [61]. Interestingly although both our structural model data and data from the measured affinity of H7 HA for human-type receptor both predicted high binding, a study recently reported that the binding of H7N9 virus to human respiratory tract was low [62]. Furthermore, another study reported that although H7N9 strains readily transmitted to naive ferrets through direct contact, replicated to higher titre in human airway epithelial cells and in the respiratory tract of ferrets and showed greater infectivity and lethality in mice compared to genetically related H7N9 and H9N2 viruses H7N9, unlike the seasonal H3N2 virus, did not transmit readily by respiratory droplets [63]. This was said to correlate with low receptor-binding specificity for human-like á2,6-linked sialyl residues [63]. Differences in findings between various H7N9 laboratory studies may be explained by differences in either H7N9 viral isolates, experimental methodologies or the glycan substrates used to measure receptor binding in these studies. The final confirmation of the accuracy of our model predictions must await resolution of these differences between H7N9 binding measurements obtained by different groups. Nevertheless, we have shown that it is possible to apply a structural modelling approach to predict the HA structure encoded by a novel influenza virus and thereby help assess its potential pandemic risk. Our structural modelling approach has the virtue that it also allows prediction of whether the new HA molecule can be neutralized by existing human antibodies. As shown, this structural modelling approach complements data from traditional sequence alignment-based approaches.

With the ever-present threat of another influenza pandemic, more surveillance centres worldwide are needed to better monitor and rapidly sequence circulating influenza viruses and identify new strains, with recent data from Chinese sentinel sites suggesting that the small number of H7N9 severe clinical cases were just a reflection of a much larger number of undetected subclinical cases [64]. Whilst they will not replace the need for formal based virus characterization, computer-based algorithms and structural modelling could be useful for performing initial screens on influenza virus variants generated by such surveillance programs. Ultimately, the power afforded by *in-silico* structural modelling approaches may allow evolutionary studies of computationally generated influenza virus variants to assist prediction of the likely future evolution of currently circulating strains. This could potentially enable vaccines to be made against future influenza viruses, as opposed to the current practice of making vaccines retrospectively against previously circulating viruses.

## Supporting Information

File S1 **Supporting figures.**
(DOCX)
