# Supplementary material for: In Silico Structural Homology Modelling and Docking for Assessment of Pandemic Potential of a Novel H7N9 Influenza Virus and Its Ability to Be Neutralized by Existing Anti-Hemagglutinin Antibodies
Source: PLoS One. 2014 Jul 21;9(7):e102618. doi: 10.1371/journal.pone.0102618 (PMC4105636; doi:10.1371/journal.pone.0102618)
Supplement: File S1 — Supporting figures. (DOCX) [file pone.0102618.s001.docx]

**Figure S1**

**
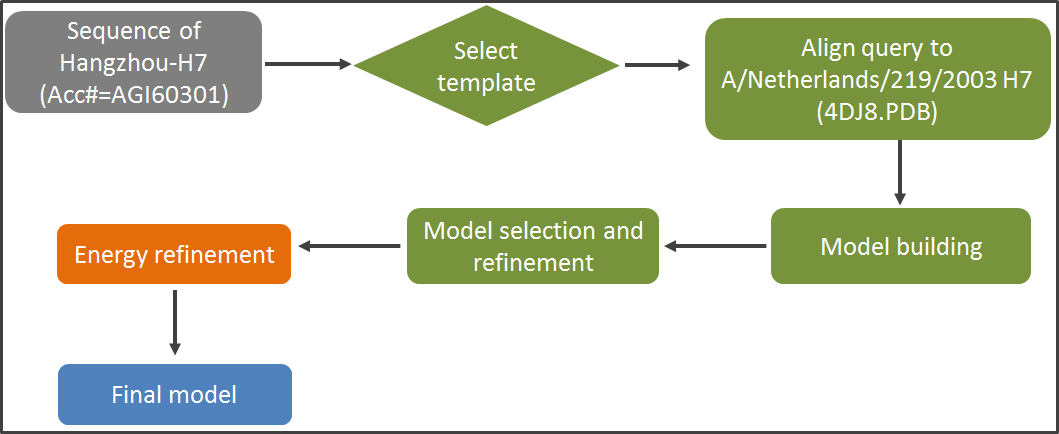
**

**Figure S1**

**Modelling method for Hangzhou-H7.** Flow diagram showing how the influenza hemagglutinin sequence Hangzhou-H7 was submitted to the Swiss-model workplace to identify suitable templates for generating a homology model for H7N9 hemagglutinin. Using the H7N7 crystal structure 4DJ8.PDB that demonstrated 96% sequence homology to Hangzhou-H7, nine homology models of Hangzhou-H7 were generated and the best model selected based on the Descreat Optimised Protein Energy Score (DPOES). After loop optimisation, the model was subjected to energy minimisation and relaxation using the molecular dynamics program NAMD resulting in the final model.

**Figure S2**

**
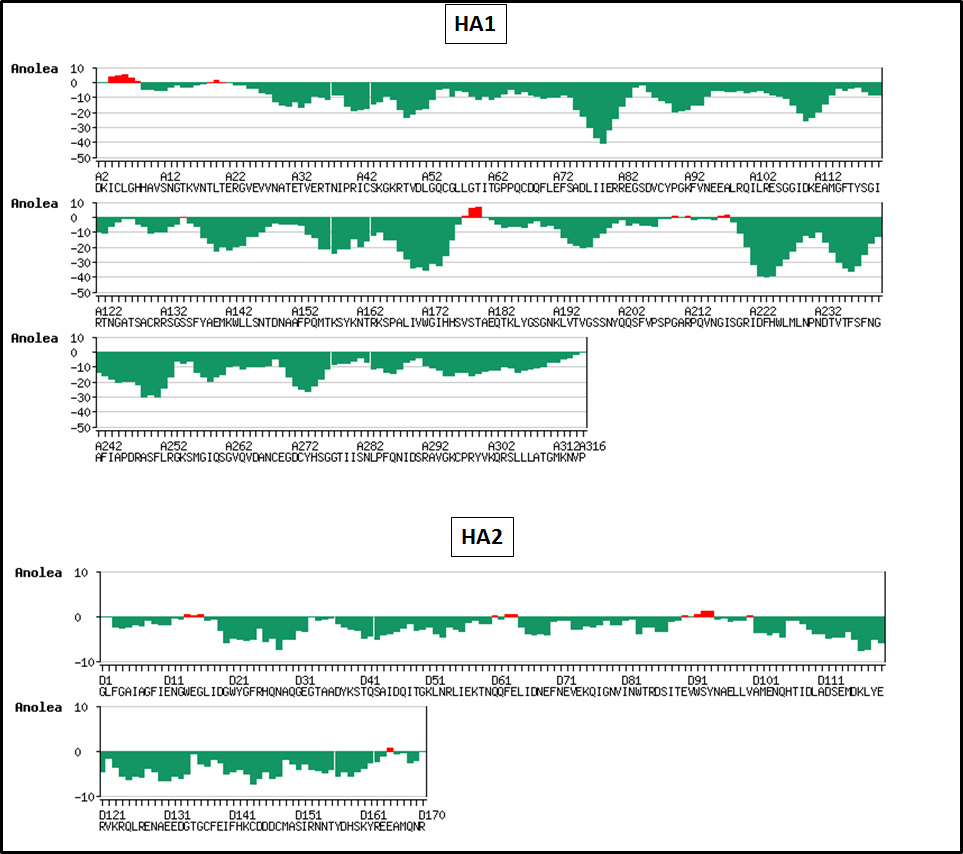
**

**Figure S2**

**Packing quality of Hangzhou-H7 structural model.** The model of Hangzhou-H7 was submitted to ANOLEA server and the packing quality was assessed for the HA1 and HA2 chains. In the figure energy values are presented as a function of the position within the amino acid sequence. Favourable negative energy values are presented in green and potentially unfavourable positive energy vales are presented in red.

**Figure S3**

**
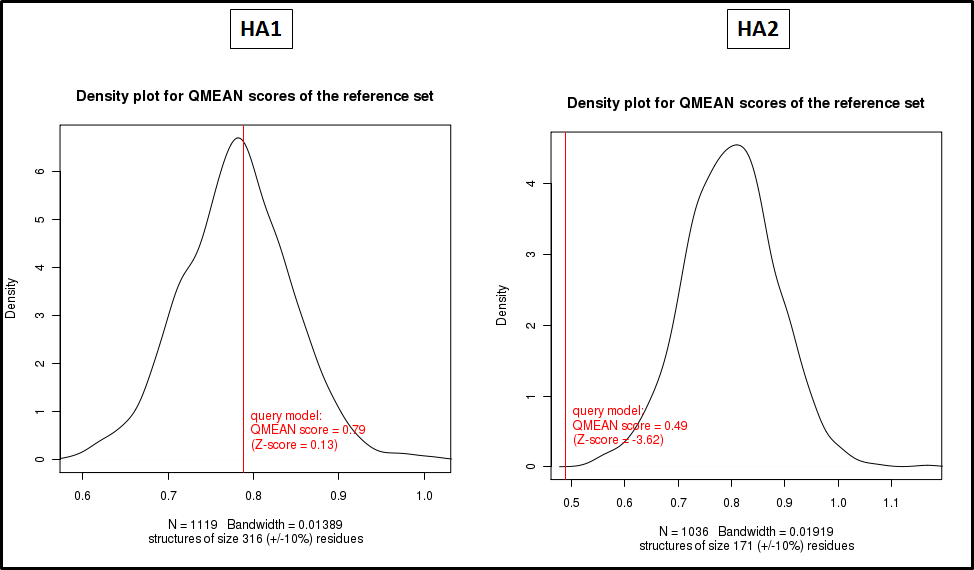
**

**Figure S3**

**QMEAN estimation of Hangzhou-H7 model quality.** Hangzhou-H7 models of HA1 and HA2 were submitted to the QMEAN server and absolute model quality was assessed. The distribution of QMEAN Z-scores for the reference X-ray crystal structures are shown by the black line and Z-score for each model by the red vertical line.

**Figure S4**

**
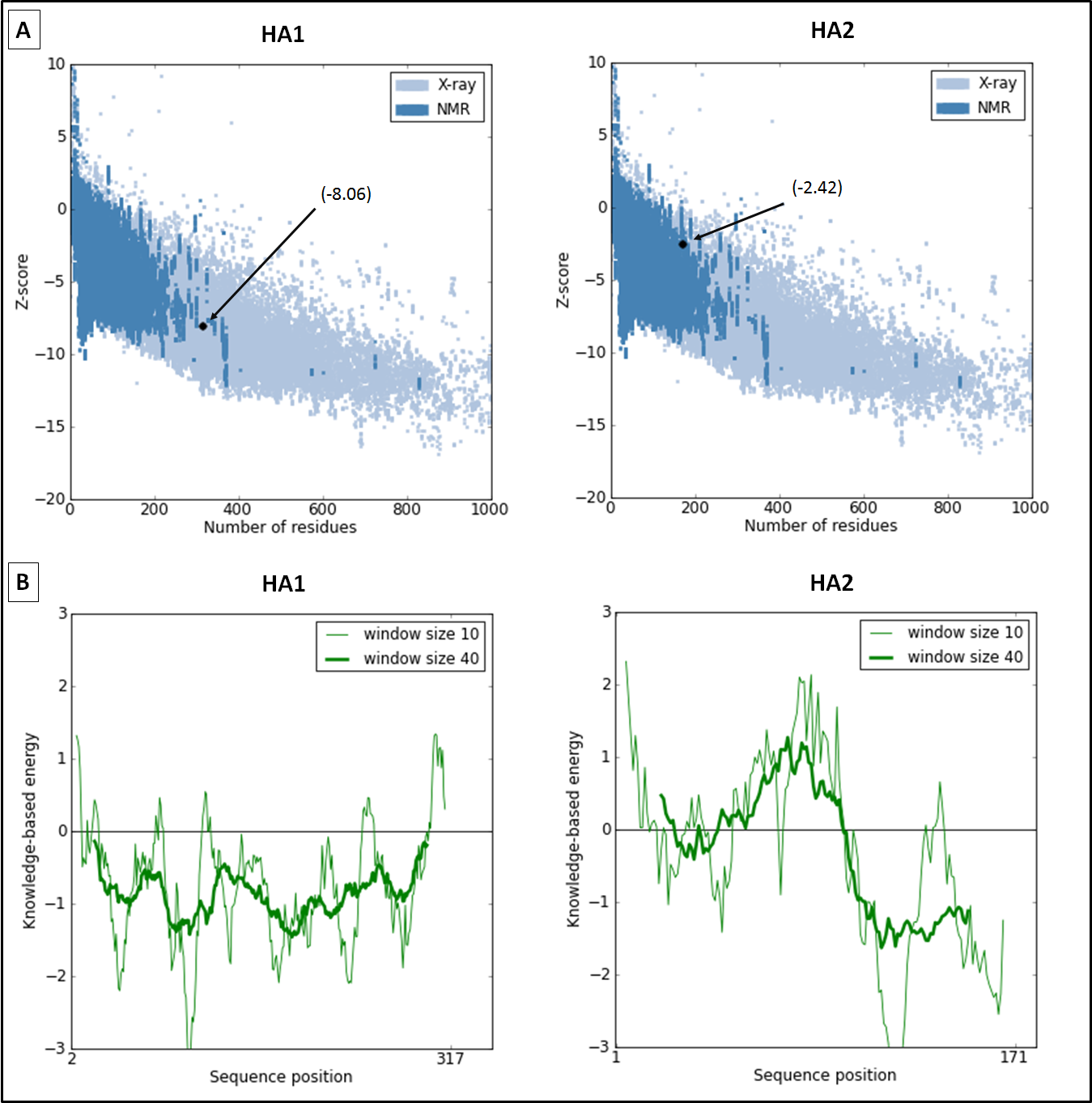
**

**Figure S4**

**ProSA validation of Hangzhou-H7 structural model.**

The homology-based structural model of Hangzhou-H7 was submitted to ProSA server and compared with the Z-scores of experimentally determined structures by X-ray diffraction or NMR. **(A)** The Z-score distribution of known X-ray crystal structures are presented in light blue and NMR structures in dark blue. The Hangzhou-H7 model Z-scores for the HA1 and HA2 chains are represented by black dots. **(B)** The energy distribution of the Hangzhou-H7 model as a function of the amino acid sequence. The more variable thin line represents the cumulative energy of 10 consecutive amino acids in the sequence and the thick line represents the cumulative energy of 40 consecutive amino acids.

**Figure S5**

**
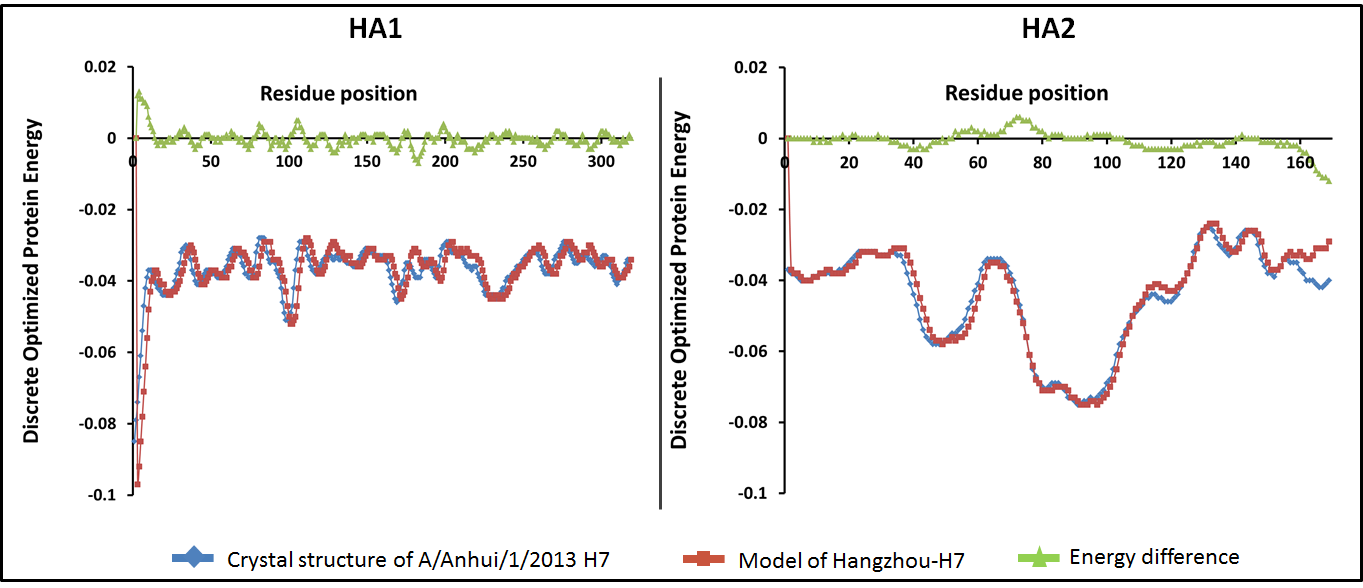
**

**Figure S5**

**Discrete Optimised Protein Energy Scores (DOPES) of the model *vs* the Crystal structure**

The DOPE scores were calculated for model of Hangzhou-H7 and the crystal structure of A/Anhui/1/2013 H7 using modeller model evaluation script. Then the scores were plotted in the same graph for comparison. The DOPES for the crystal structure is presented by the blue line, the maroon line presents the DOPES of the model of Hangzhou-H7. The green line presents the difference between the DOPES of the crystal structure and the model.

**Figure S6**

**
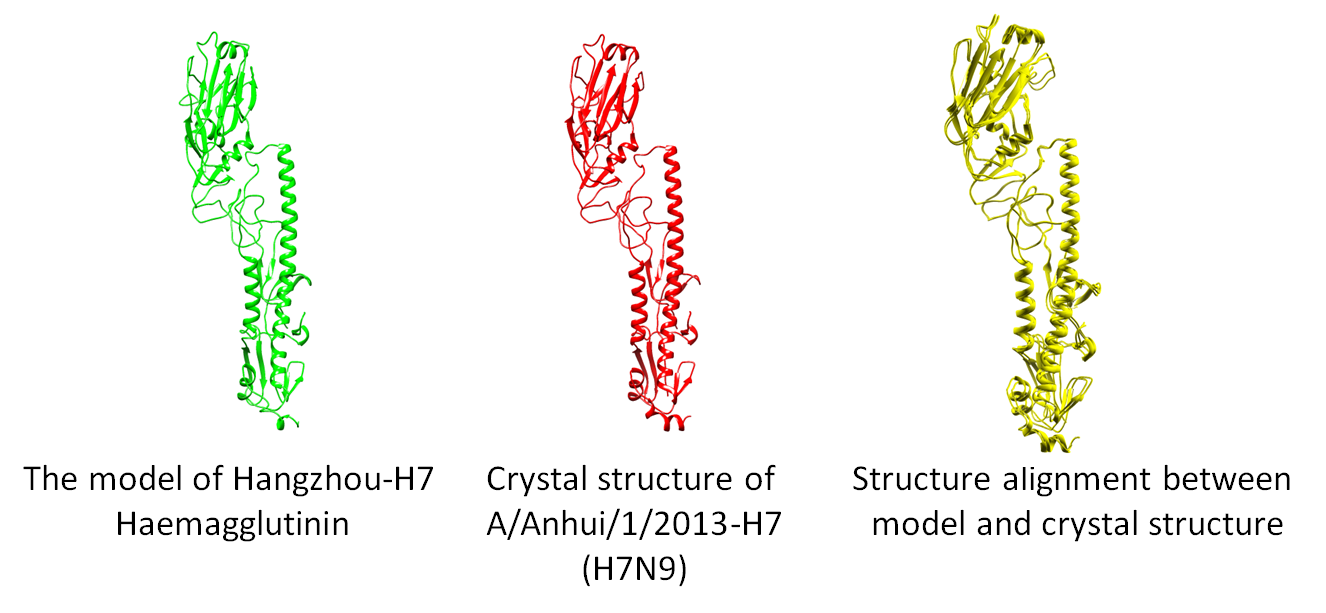
 Figure S6**

**Comparison between the model and the crystal structure of H7N9 hemagglutinin**The model of Hangzhou-H7 constructed using only the sequence is highlighted in green and the crystal structure of A/Anhui/1/2013 H7 is highlighted in red. In the right, the over lay of the crystal structure and the model is highlighted in yellow. RMSD for the structure alignment is 0.721 Å.
